# Of Retinoids and Organotins: The Evolution of the Retinoid X Receptor in Metazoa

**DOI:** 10.3390/biom10040594

**Published:** 2020-04-11

**Authors:** Elza Fonseca, Raquel Ruivo, Débora Borges, João N. Franco, Miguel M. Santos, L. Filipe C. Castro

**Affiliations:** 1 Interdisciplinary Centre of Marine and Environmental Research, University of Porto, 4450-208 Matosinhos, Portugal; fonseca.ess@gmail.com (E.F.); ruivo.raquel@gmail.com (R.R.); debora.borges@ciimar.up.pt (D.B.); joaonunofranco@gmail.com (J.N.F.); 2 MARE—Marine and Environmental Sciences Centre, ESTM, 2520-637 Peniche, Portugal; 3 Department of Biology, Faculty of Sciences, University of Porto, 4169-007 Porto, Portugal

**Keywords:** retinoid X receptor, metazoa, Bryozoa, endocrine disruption, 9-cis retinoic acid, TBT

## Abstract

Nuclear receptors (NRs) are transcription factors accomplishing a multiplicity of functions, essential for organismal homeostasis. Among their numerous members, the retinoid X receptor (RXR) is a central player of the endocrine system, with a singular ability to operate as a homodimer or a heterodimer with other NRs. Additionally, RXR has been found to be a critical actor in various processes of endocrine disruption resulting from the exposure to a known class of xenobiotics termed organotins (e.g., tributyltin (TBT)), including imposex in gastropod molluscs and lipid perturbation across different metazoan lineages. Thus, given its prominent physiological and endocrine role, RXR is present in the genomes of most extant metazoan species examined to date. Here, we expand on the phylogenetic distribution of RXR across the metazoan tree of life by exploring multiple next-generation sequencing projects of protostome lineages. By addressing amino acid residue conservation in combination with cell-based functional assays, we show that RXR induction by 9-cis retinoic acid (9cisRA) and TBT is conserved in more phyla than previously described. Yet, our results highlight distinct activation efficacies and alternative modes of RXR exploitation by the organotin TBT, emphasizing the need for broader species sampling to clarify the mechanistic activation of RXR.

## 1. Introduction

Nuclear receptors (NRs) constitute an exceptionally vast family of metazoan transcription factors [1,2]. Being mostly ligand-activated, NRs play pivotal roles in cellular endocrine processes, including metabolism, development, and reproduction [3,4]. Of such, the retinoid X receptor (RXR, NR2B) is particularly unique. Given its ability to operate as a homodimer or as a heterodimer with other NRs (i.e., retinoic acid receptor (RAR, NR1B), peroxisome proliferator-activated receptor (PPAR, NR1C), and thyroid hormone receptor (THR, NR1A)] [5,6,7,8,9], RXRs participate in intricate networks of cellular functions impacting the overall organismal homeostasis [10,11]. Accordingly, metazoan RXRs have been acknowledged not only as critical players for the maintenance of normal physiological states but also as prime targets of disruption by exogenous compounds [12,13,14].

The ongoing explosion in the assembly of full genome sequences from multiple species/lineages is key to understand NR gene diversification. RXR, for instance, has been found in the genomes of most extant metazoan species examined to date [15,16]. Nevertheless, the lack of data, especially from invertebrate protostomes, and the poor taxonomic sampling of most studies can lead to erroneous interpretations [17]. Moreover, the presence of a receptor per se does not warrant a similar molecular/physiological role, and various observations have hinted that ligand–receptor pairs may not always be stable in evolutionary time scales, exhibiting changes in binding specificities and activation profiles [18,19,20]. RXR, for instance, is activated by small lipophilic molecules, including the high-affinity ligand 9-cis retinoic acid (9cisRA) [21]. This canonical high affinity activity has been corroborated in various lineages such as vertebrates, molluscs, and annelids [22,23,24,25,26,27,28]. In the cephalochordate *Branchiostoma floridae*, on the other hand, 9cisRA binding is maintained, albeit with lower affinity [29]. Within ecdysozoans, several recognition and activation patterns have been identified, with clear differences even within lineages. For example, while most insect RXR homologues, named ultraspiracle (USP), are unresponsive to 9cisRA [30,31], *Locusta migratoria* USP retains high affinity towards 9cisRA and all-trans retinoic acid (ATRA) [32]. Regarding crustaceans, 9cisRA binding has been suggested, as exemplified by the *Daphnia magna* RXR, not always yielding gene transcription [33]. However, the physiological relevance of 9cisRA is still debated. In fact, low amounts of endogenous 9cisRA are usually detected in animal tissues, and this is further hampered by the instability of RA isomers [34]. Moreover, other molecules, such as polyunsaturated fatty acids (PUFAs), were also suggested to act as natural ligand of RXR [35,36]. Thus, the full set of endogenous RXR ligands remains to be fully determined [21,37].

Besides natural ligands, RXRs have also been shown to be exploited by endocrine-disrupting chemicals (EDCs), which are substances with the potential to alter endocrine functions causing physiological imbalances, leading to developmental, reproductive, and metabolic defects [38,39,40]. The organotins tributyltin (TBT) and triphenyltin (TPT) were extensively used as biocides in anti-fouling paints, being now persistent organic pollutants in aquatic environments [41]. These compounds serve as a significant example, with TBT-dependent activation of gastropod mollusc RXRs suggested as the prime cause of imposex development [24,26]. TBT ability to induce RXR-dependent transcription was demonstrated in human, mollusc, annelid, and crustacean RXRs and seems reliant on the presence of a specific cysteine residue within the ligand-binding domain (LBD) [24,27,33,42,43,44].

Despite the significant wealth of NR evolutionary research, key phyla such as Brachiopoda, Rotifera, Bryozoa, Phoronida, Priapulida, Kinorhyncha, and many others remain uncharacterized. Here, we expand our knowledge on the phylogenetic distribution of RXR across the metazoan tree of life. Furthermore, we provide functional characterization of RXRs from representative species of still unexplored lineages and their susceptibility to endocrine disruption by TBT.

## 2. Materials and Methods

### 2.1. Sampling and RNA Extraction

*Bugula neritina* specimens were collected at the Marina da Póvoa de Varzim, Portugal; *Phoronopsis californica*, and *Megathiris detruncata* specimens were collected at Madeira Island, Portugal; *Bonellia viridis* was sampled at Peniche, Portugal; *Priapulus caudatus* and *Xenoturbella bocki* specimens were collected at the Gullmarn fjord, Sweden. The specimens were preserved on RNAlater (Invitrogen, Carlsbad, CA, USA) immediately after their collection for further total RNA extraction. A small portion of each specimen was homogenized with PureZOL RNA Isolation Reagent^®^ (Bio-Rad, Hercules, CA, USA), and chloroform was used to extract the nucleic acids according to the manufacturer’s instructions. The illustra RNAspin Mini RNA Isolation (GE Healthcare, Chicago, IL, USA) kit was used to isolate the total RNA from the aqueous phase obtained in the first step. and genomic contamination was prevented by an on-column DNAse I digestion step. RNA was eluted in 30 μL of RNase-free water. The synthesis of first-strand cDNA (1 μg) and of 5’and 3´ cDNA for rapid amplification of cDNA ends (RACE) was performed with the iScript™cDNA Synthesis Kit (Bio-Rad, Hercules, CA, USA) and the SMARTer™ RACE cDNA Amplification kit (Clontech, Mountain View, CA, USA), respectively, following to the manufacturer’s guidelines.

### 2.2. RXR Gene Isolation

The open reading frames (ORF) of *RXR* genes were obtained by a combination of RACE, nested, hemi-nested, and/or degenerated polymerase chain reactions (PCRs), using Phusion Flash High-Fidelity PCR Master Mix (Thermo Fisher Scientific Co., Waltham, MA, USA) according to the manufacturer’s protocol. Partial gene sequence of *B. viridis RXR* and *B. neritina RXR1* and *RXR2* and two partial *RXR*-like sequences from *X. bocki* were retrieved through a BLAST approach using Sequence Read Archive (SRA) files. A partial gene sequence of *P. californica RXR* and *M. detruncata RXR* were obtained by PCR with degenerated primers (Supplementary Material Table S1) designed with the CODEHOP software [45]. The sequences obtained with degenerated PCRs and BLAST searches were used to design specific primers (Supplementary Material Table S1) with the Primer3 (v.0.4.0) software [46,47]. All PCR products were purified using NZYGelpure (Nzytech, Lisbon, Portugal), cloned into Nzy5α competent cells (Nzytech, Lisbon, Portugal) using the pGEM-T Easy Vector System (Promega, Madison, WI, USA), and the sequences were confirmed by sequencing (Eurofins GATC, Constance, Germany). The sequences have been deposited in GenBank (Accession numbers: *P. californica RXR* MT264997, *M. detruncata RXR* MT264998, *B. viridis RXR* MT264999, *B. neritina RXR1* MT265000, *B. neritina RXR2* MT265001, and *X. bocki RXR* MT265002). *Trichoplax adhaerens* and *Aurelia aurita RXR* ORFs (Table 1) were chemically synthetized by NZYTech (Lisbon, Portugal).

### 2.3. Sequence and Phylogenetic Analysis

The amino acid sequences from the isolated *RXR* genes were aligned with human RAR, HNF4, COUP and RXR amino acid sequences and RXR amino acid sequences from other metazoans retrieved from the GenBank database and blast researches on SRA files (Table 1). The full amino acid sequences were aligned with the Multiple Alignment using Fast Fourier Transform (MAFFT) server (v.7) [48], using the L-INS-i method. The alignment with 43 sequences and 1133 positions was used in a Bayesian phylogenetic analysis with MrBayes (v.3.2.3) sited in the CIPRES Science Gateway (v.3.3) [49]. The following parameters were used: generation number = 10,000,000, rate matrix for aa = mixed (Jones), nruns = 2, nchains = 4, temp = 0.20, sampling set to 1000, and burnin to 0.25. The statistical support for each branch was expressed as Bayesian posterior probabilities [50] and indicated at the nodes. FigTree (v.1.3.1) was used to visualize the tree. The previous alignment was visualized and edited in Geneious^®^ (v7.1.7) to identify the residues known to interact with 9cisRA and TBT, based on previous studies [29,42,51,52,53].

### 2.4. Construction of Plasmid Vectors

The hinge and LBD regions of *H. sapiens RXRα*, *P. caudatus RXR*, *T. adhaerens RXR*, *A. aurita RXR*, and of the isolated *RXRs* were amplified by PCR using specific primers (Supplementary Material Table S2) and Phusion Flash High-Fidelity PCR Master Mix (Thermo Fisher Scientific Co., Waltham, MA, USA), according to the protocol from the supplier. The amplicons were then digested with restriction enzymes (Supplementary Material Table S2) and ligated to the pBIND plasmid (AF264722; Promega, Madison, WI, USA) with T4 ligase (Promega, Madison, WI, USA) which expresses the *Renilla reniformis* luciferase and contains a yeast Gal4 DNA-binding domain (DBD) that acts on an upstream activation sequence (UAS) response element to produce GAL4 DBD–RXR LBD “chimeric” receptors. The sequences of the plasmid constructs were confirmed by sequencing (Eurofins GATC, Constance, Germany). The *B. neritina* mutants RXR1 (Thr388Cys), RXR2 (Met231Ala), RXR2 (Val294Ala), and RXR2 (∆Ser211-Asp227) were produced by NZYTech (Lisbon, Portugal).

### 2.5. Chemicals and Solutions

The compounds 9cisRA, TBT chloride, and sterile-filtered dimethyl sulfoxide (DMSO) were obtained from Sigma-Aldrich (St. Louis, MO, USA). The stock solutions were prepared in DMSO: 9cisRA at 1 mM, TBT at 10, 100, and 250 μM.

### 2.6. Cell Culture and Transactivation Assays

Cell culture and transactivation assays were performed as previously described in [27,54,55]. Briefly, Cos-1 cells (Sigma-Aldrich) were maintained at 37 °C with 5% CO_2_ (humidified atmosphere) in Dulbecco’s modified Eagle’s medium (DMEM) (PAN-Biotech, Aidenbach, Bayern, Germany) supplemented with 10% and 1% of fetal bovine serum (PAN-Biotech, Aidenbach, Bayern, Germany) and penicillin/streptomycin (PAN-Biotech, Aidenbach, Bayern, Germany), respectively. Cells were seeded in 24-well culture plates and transfected after 24 h with 0.5 μg of one of the GAL4 DBD–RXR LBD constructs and 1 μg of pGL4.31 [luc2P/ GAL4UAS/Hygro] luciferase reporter vector (Promega, Madison, WI, USA), which contains five UAS elements upstream of the firefly luciferase reporter gene, using lipofectamine 2000 reagent (Invitrogen, Carlsbad, CA, USA) in Opti-MEM (Gibco, Carlsbad, CA, USA), according to the manufacturer’s indications. After 5 h of incubation, the transfection medium was replaced with medium containing 9cisRA (1 μM) or TBT (10, 100, and 250 nM) dissolved in DMSO (0.1%). Cells were lysed 24 h after transfection. The Dual-Luciferase Reporter Assay System (Promega, Madison, WI, USA) was used to assay firefly luciferase (reporter pGL4.31) and *Renilla* luciferase (pBIND) activities following the manufacturer’s instructions. All transfections were performed with two technical replicates per condition in three independent assays. The results are expressed as the fold induction calculated as the ratio between firefly luciferase (reporter pGL4.31) and *Renilla* luciferase (internal control for transfection efficiency) and normalized by dividing by the control (DMSO) (firefly luciferase/*Rennila* luciferase ratio datasets in Supplementary Material Table S3).

### 2.7. Statistical Analysis

The differences between the mean of the technical replicates were tested for significance using Student’s t-test and one-way analysis of variance (ANOVA), followed by the Holm–Sidak test. Data were transformed whenever the homogeneity of variances and/or the normality failed. The level of significance (*P*-value) was set to 0.05. Statistical analysis was performed using SigmaPlot software (v.11.0).

## 3. Results

### 3.1. Phylogenetic and Sequence Analyses of RXR in Metazoan Lineages

**Figure 1:**
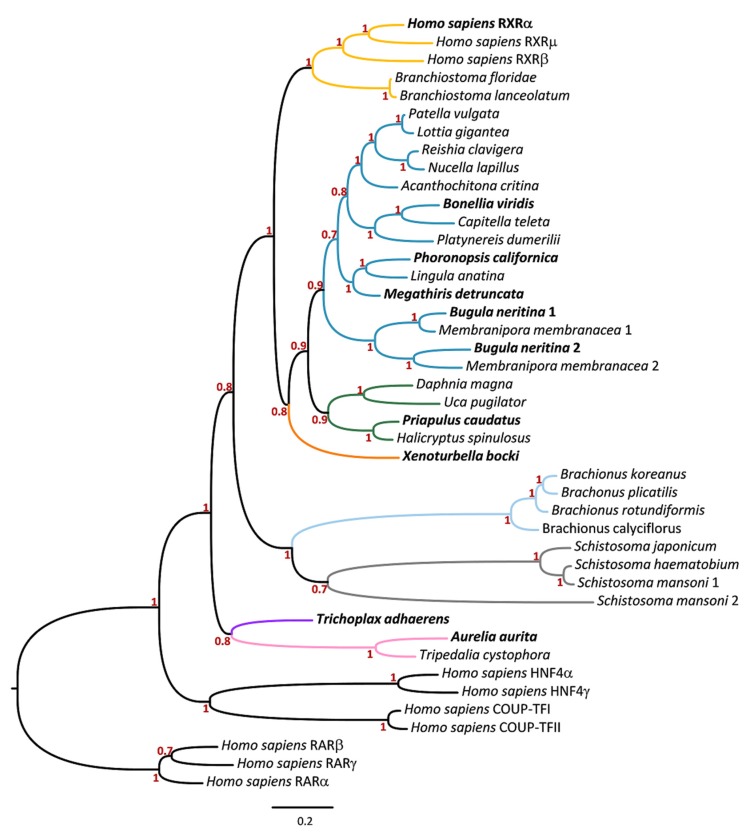
Bayesian analysis of the phylogenetic distribution of RXR among metazoan lineages. Chordata (yellow); Lophotrochozoa (blue): Mollusca, Annelida, Phoronida, Brachiopoda, and Bryozoa, Platyhelminthes (grey), Rotifera (light blue); Ecdysozoa (green): Arthropoda and Priapulida; Xenoturbellida (orange); Placozoa (purple); Cnidaria (pink); numbers at nodes indicate Bayesian posterior probabilities. Human retinoic acid receptor (RAR) amino acid sequences were used as outgroup to root the tree. The RXRs used in the subsequent functional analysis are highlighted in bold.

In the present study, we investigated a set of currently available genomes and transcriptomes from multiple metazoan phyla to retrieve partial RXR amino acid sequences. By combining database mining with PCR approaches, we were able to successfully isolate full-length *RXR* ortholog genes from several species, validating the occurrence of *RXRs* within the selected Metazoa lineages (Table 1). Phylogenetic analysis robustly corroborated the orthology of the new isolated *RXR* genes (Figure 1). For most newly examined species, a single *RXR* ortholog gene was retrieved. A notable exception were bryozoans (*B. neritina* and *M. membranacea*), for which two *RXR* genes were identified (Figure 1).

**Figure 2:**
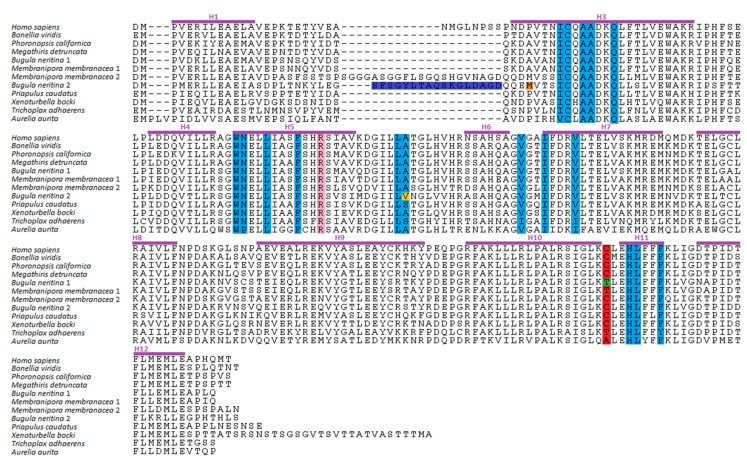
Alignment of RXR ligand-binding domains (LBD) from *Homo sapiens*, *Bonellia viridis*, *Phoronopsis californica*, *Megathiris detruncata*, *Bugula neritina*, *Membranipora membranacea*, *Priapulus caudatus*, *Xenoturbella bocki*, *Trichoplax adhaerens*, and *Aurelia aurita*. The α-helices from the human RXRα secondary structure are indicated with purple lines. The residues forming the pocket in human RXRα are highlighted in blue. The arginine residue that forms an ionic interaction with 9cisRA is highlighted in pink. The cysteine residue that forms a covalent bond with tributyltin (TBT) is highlighted in red. The *B. neritina* mutants RXR1 (Thr388Cys), RXR2 (Met231Ala), RXR2 (Val294Ala), and RXR2 (∆Ser211-Asp227) are highlighted in green, orange, yellow, and dark blue, respectively.

The amino acid sequence alignment of the LBD of RXRs revealed that the 18 amino acid residues located in the α-helices H3, H5, H7, and H11 and in the β-turn of the LBD, which confer the ability of binding to 9cisRA and induce a transcriptional response in human RXRα [51,52], are overall conserved in the new isolated RXRs (Figure 2), suggesting that these receptors could be able to interact with 9cisRA. The exceptions were RXR2 from *B. neritina* (Bryozoa), exhibiting one non-conserved residue, and the RXRs from *P. caudatus* (Priapulida), *T. adhaerens* (Placozoa), and *A. aurita* (Cnidaria), with one, four, and three non-conserved residues, respectively (Figure 2). On the other hand, the cysteine residue in helix H11, crucial for transactivation of human RXRα by organotins (TBT and TPT) [42,56], is substituted in bryozoan (*B. neritina* and *M. membranacea*) RXR1s, cnidarian (*A. aurita*), and placozoan (*T. adhaerens*) RXRs, suggesting a possible inability to respond to such compounds. In addition, and unlike other invertebrate RXRs, 17 to 21 amino acid insertions were also found in *B. neritina* and *M. membranacea* RXR2s, respectively.

### 3.2. In vitro Interaction of RXRs with 9cisRA

**Figure 3:**
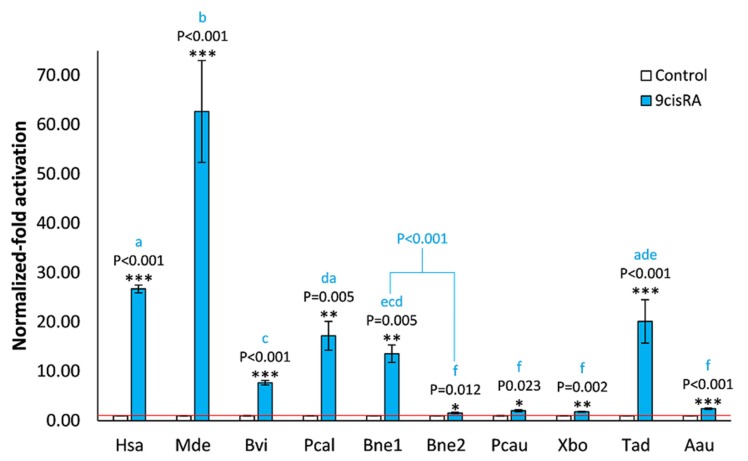
Transactivation activity of luciferase reporter gene mediated by GAL4 DNA-binding domain (DBD)–RXR LBD chimeric receptors in COS-1 cells in the presence of 9-cis retinoic acid (9cisRA). Data represent means ± SEM from three independent experiments (n = 3). The results were normalized to the control condition (DMSO without ligand). Significant differences between 9cisRA and the solvent control were inferred with Student’s t-test. Asterisks denote significant differences (* *P* ≤ 0.05, ** *P* ≤ 0.01, *** *P* ≤ 0.001). Significant differences among the different RXRs in the test condition were inferred with one-way ANOVA (source of variation: F = 61.9, *P* < 0.001). The different letters denote significant differences. Hsa, *H. sapiens*; Mde, *M. detruncata*; Bvi, *B. viridis*; Pcal, *P. californica*; Bne, *B. neritina*, Pcau, *P. caudatus*; Xbo, *X. bocki*; Tad, *T. adhaerens*; Aau, *A. aurita*.

Since the main residues of the human RXRα pocket known to interact with 9cisRA are conserved in most of the isolated RXRs, we tested their ability to activate gene expression upon binding to 9cisRA, using a luciferase reporter gene assay (Figure 3). Our results showed a significant activation (*P* < 0.05) of the chimeric RXRs in the presence of 1 μM 9cisRA, yielding weaker activations than the human RXRα, with the exceptions of the phoronid *P. californica* (*P* = 0.073) and the placozoan *T. adhaerens* (*P* = 0.176) RXRs. Unexpectedly, the brachiopod *M. detruncata* RXR elicited an activation stronger than the human control (*P* = 0.002). Also, among the analyzed RXRs, low affinity towards 9cisRA was verified with respect to the bryozoan *B. neritina* RXR2, the priapulid *P. caudatus*, the xenoturbellid *X. bocki,* and the cnidarian *A. aurita* RXRs. Interestingly, the efficacy/effectiveness of 9cisRA was significantly different (*P* < 0.001) for the bryozoan RXRs.

### 3.3. In vitro Interaction of RXRs with TBT

**Figure 4:**
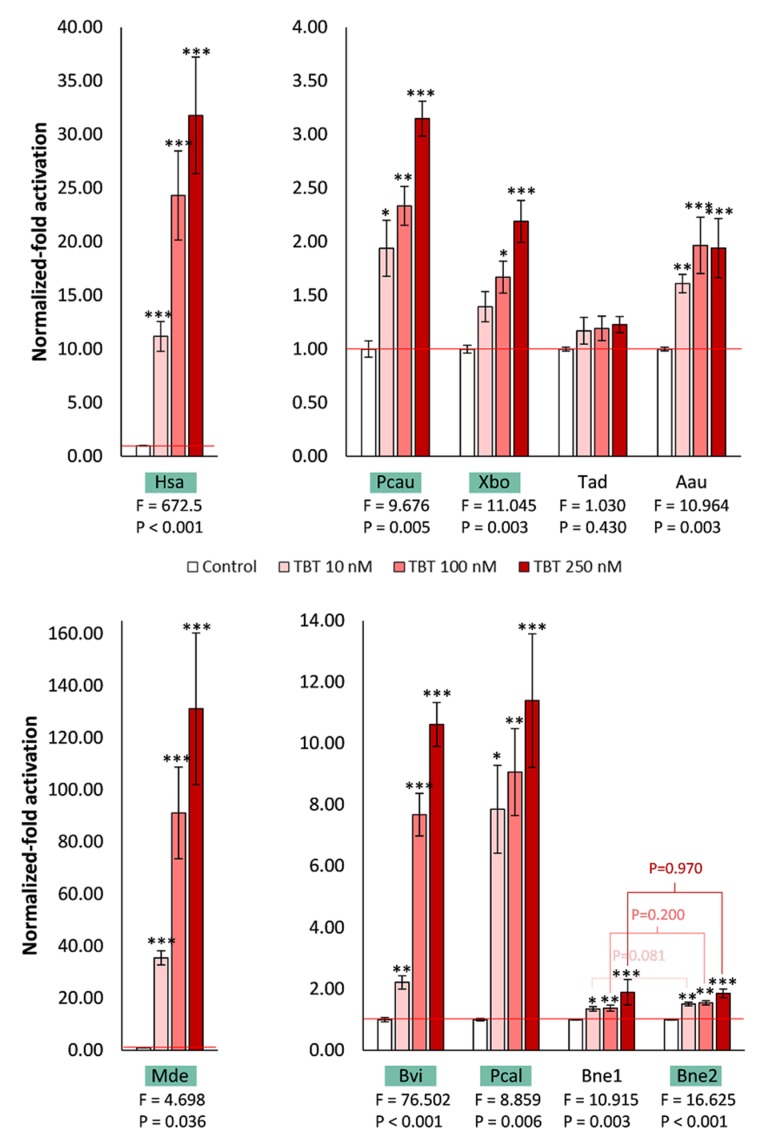
Transactivation activity of the luciferase reporter gene mediated by GAL4 DBD–RXR LBD chimeric receptors in COS-1 cells in the presence of TBT. Data represent means ± SEM from three separate experiments (n = 3). The results were normalized to the control condition (DMSO without ligand). Significant differences between the tested concentrations of TBT and the solvent control were inferred with one-way ANOVA. The F and *P* values of the source of variation are indicated below each species name abbreviation. Asterisks denote significant differences (* *P* ≤ 0.05, ** *P* ≤ 0.01, *** *P* ≤ 0.001). Significant differences between BneRXR1 and BneRXR2 with TBT at the same concentration were inferred with Student’s t-test. The green rectangles indicate the RXRs which have the cysteine residue previously described as crucial for binding to TBT. Hsa, *H. sapiens*; Mde, *M. detruncata*; Bvi, *B. viridis*; Pcal, *P. californica*; Bne, *B. neritina*, Pcau, *P. caudatus*; Xbo, *X. bocki*; Tad, *T. adhaerens*; Aau, *A. aurita*.

As classical targets of endocrine disruption by organotins [24,26,38,39], we tested these RXRs for their ability to be activated by TBT and to promote the expression of the luciferase reporter gene (Figure 4). With the exception of the placozoan *T. adhaerens* RXR, all the tested RXRs were able to significantly (*P* < 0.05) activate gene expression, at least at the higher tested concentrations of TBT (100 and 250 nM). Once again, we found a stronger response for the brachiopod *M. detruncata* RXR when compared to the human RXRα, and weaker responses for the remaining receptors. Even without conservation of the previously described cysteine residue, suggested to be key for TBT binding and mediation of transactivation [42,56], the cnidarian *A. aurita* RXR and the bryozoan *B. neritina* RXR1 were able to promote gene transcription in the presence of TBT, similarly to the rotifer *B. koreanus* RXR [40]. Curiously, and despite the conservation of the key cysteine residue in the bryozoan *B. neritina* RXR2, both subtypes responded similarly to TBT (*P* > 0.05), indicating that TBT possibly interacts with the pocket environment of both receptors in a similar fashion.

### 3.4. Mutagenesis of Bryozoan Specific RXR Orthologs

**Figure 5:**
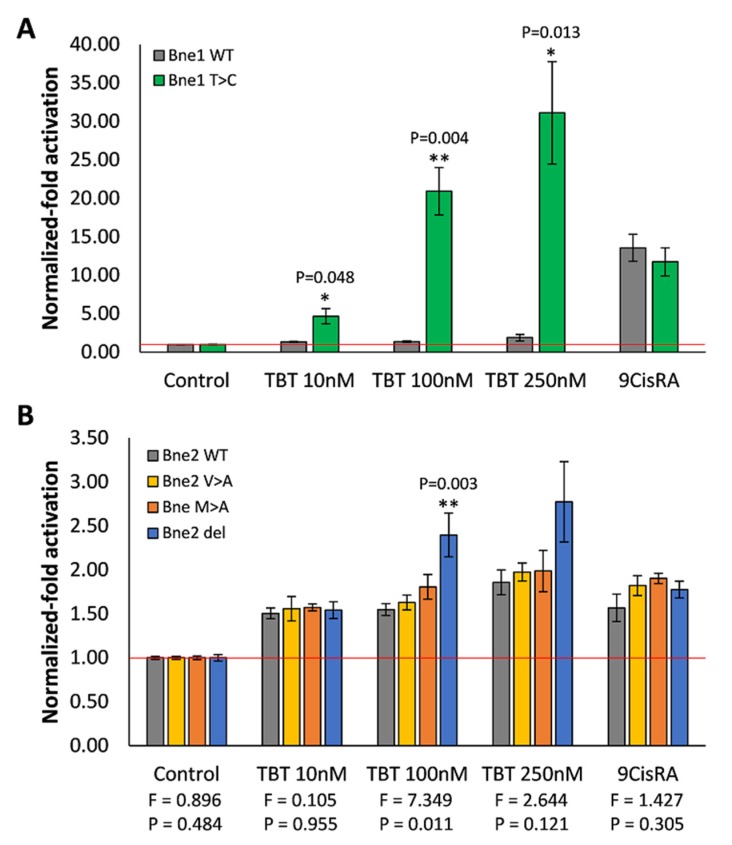
Transactivation activity of the luciferase reporter gene mediated by *B. neritina* RXRs in COS-1 cells in the presence of 9cisRA and TBT. **A** *B. neritina* RXR1 wild-type (Bne1 WT) versus *B. neritina* RXR1 mutant Thr388Cys (Bne2 T > C); **B** *B. neritina* RXR2 wild-type (Bne2 WT) versus *B. neritina* RXR2 mutants Met231Ala (Bne2 M > A), Val294Ala (Bne2 V > A), and ∆Ser211–Asp227 (Bne2 del). Data represent means ± SEM from three separate experiments (n = 3). The results were normalized to the control condition (DMSO without ligand). In each condition, the significant differences between *B. neritina* RXR1 wild-type and mutant and between *B. neritina* RXR2 wild-type and mutants were inferred with Student’s t-test and one-way ANOVA, respectively. The F and *P* values of the source of variation for each ANOVA analysis are indicated below each group condition. Asterisks denote significant differences (* *P* ≤ 0.05, ** *P* ≤ 0.01).

To further clarify the transcriptional responses obtained with the bryozoan *B. neritina* RXRs, with both 9cisRA and TBT, site-directed and deletion mutants were tested (Figure 5). First, the bryozoan RXR1 Thr388Cys mutant (Bne1 T > C) was assayed for its ability to elicit a stronger response to TBT, when compared to the wild-type receptor (Bne1 WT) (Figure 5A). In effect, the replacement of the threonine residue (Thr) by the classical key cysteine residue (Cys) prompted a significant higher response to all the tested concentrations of TBT, without affecting the response to 9cisRA. Next, using the bryozoan RXR2, three mutants were produced: the RXR2 Met231Ala (Bne2 M > A) and RXR2 Val294Ala (Bne2 V > A) mutants were tested expecting higher responses to 9cisRA, since the stereochemistry of Bne2M > A and Bne2V > A mutants was expected to be more similar to the one of RXR1, while the RXR2 ∆Ser211–Asp227 (Bne2 del) mutant was tested expecting a favorable conformational change in the receptor binding pocket, affecting interactions to both 9cisRA and TBT (Figure 5B). A slightly higher sensitivity to 9cisRA was detected for Bne2 M > A, Bne2 V > A, and Bne2 del mutants, however, the increase was not significant (*P* > 0.05). Regarding the interaction with TBT, the Bne2 del mutant presented the highest levels of activation, being indicative that, apart from the cysteine residue, the whole structural conformation of the LDB can affect the responsiveness to TBT.

## 4. Discussion

**Figure 6:**
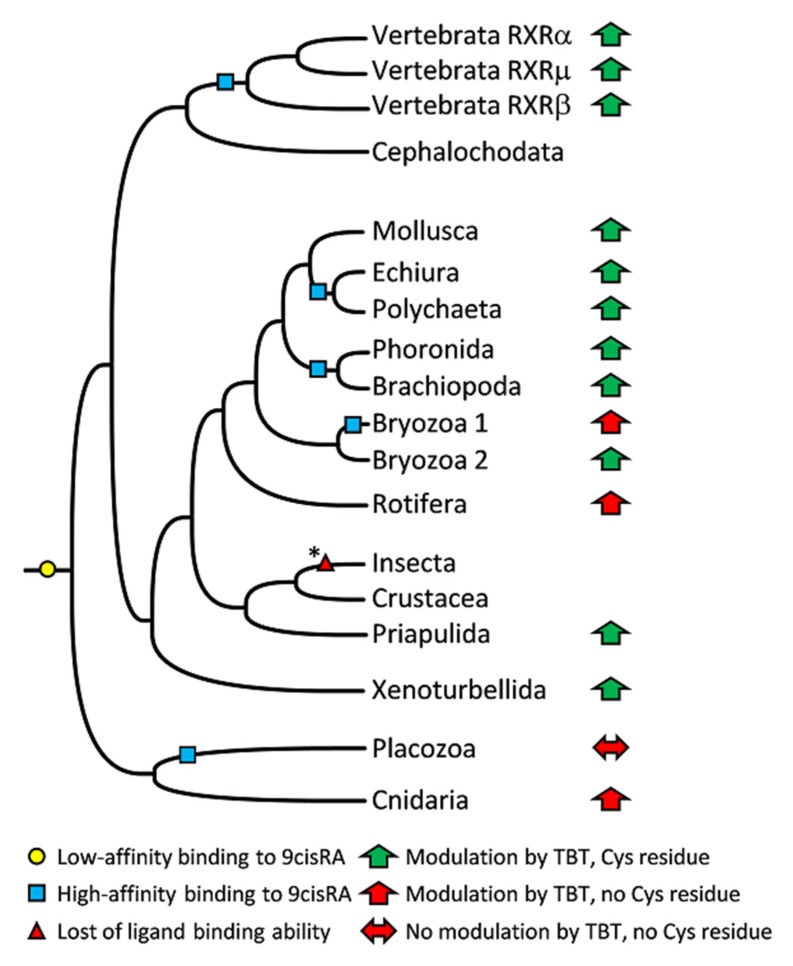
Evolutionary conservation of RXR modulation by 9cisRA and tributyltin. * The ultraspiracle (USP) ortholog from the earlier branching orthopteran *Locusta migratoria* retains high affinity towards 9cisRA and all-trans retinoic acid.

In this work, we successfully isolated full-length *RXR* gene orthologs in all of the examined metazoan species. These covered an ample set of phyla, configuring the conclusion that *RXR* is largely conserved throughout Metazoa and likely represents an essential gene for animal homeostasis and development [10,11]. Curiously, we were also able to deduce the occurrence of two *RXR* genes in bryozoan species (*B. neritina* and *M. membranacea*). Multiple RXR isoforms were previously identified in other invertebrate species such as gastropod molluscs [25,43], the platyhelminth parasite *Schistosoma mansoni* [57,58], and the bivalve *Chlamys farreri* [59]; yet, the physiological consequences of this novelty remain unknown and should be investigated in the future. The inspection of the LBD amino acid alignment from the isolated RXRs puts into perspective the possibility of these RXRs to be activated upon binding to 9cisRA, despite the substitution of some residues reported as important for the interaction with 9cisRA [51,52,53]. In fact, some studies demonstrated that the strict conservation of the residues lining the ligand-binding pocket is not sufficient to guarantee ligand–receptor interaction, as demonstrated by the insect *Tribolium castaneum* USP, which is strongly conserved but does not bind to 9cisRA [31]. On the other hand, amino acid substitutions within the RXR ligand-binding pocket from the placozoan *T. adhaerens* or the cnidarian *T. cystophora*, closely related to *A. aurita*, did not impair 9cisRA binding [14,47,51]. In the analyzed species, 9cisRA was able to induce RXR-mediated transcription, albeit with different effectiveness. With the exception of the brachiopod *M. detruncata* RXR, all examined RXRs yielded weaker responses when compared with the human homologue. These results are in accordance with previous studies of RXRs from the cephalochordate *B. floridae* [29], the molluscs *Biomphalaria glabrata* [25] and *Reishia clavigera* [60], and the annelid *Platynereis dumerilii* [27]. Specifically, in bryozoans both RXRs presented different sensitivities towards 9cisRA, similarly to the alternative splicing isoforms found in gastropod molluscs [43,60]. This finding might indicate that the two RXRs of this lineage should have different endogenous ligands and/or different physiological functions. Overall, these results further clarify the previously proposed evolutionary history of RXR, in which an ancestral function of RXR able to bind 9cisRA was improved in vertebrates and secondarily lost at the base of the insects [29]. Yet, regarding insects, and given that the earlier branching orthopteran *L. migratoria* [61] USP was suggested to bind with high affinity to 9cisRA and ATRA [32], unlike other analyzed insect USPs [30,31], a more detailed phylogenetic functional mapping should be performed to resolve episodes of loss and gain of function towards 9cisRA. Still, the present results reinforce the hypothesis of an ancestral RXR with low affinity for 9cisRA and, in addition to vertebrates, we propose an increase of affinity for 9cisRA in Brachiopoda, Phoronida, Bryozoa, Annelida, and Placozoa (Figure 6).

In addition to binding to 9cisRA, RXR has been described as a target of endocrine disruption mediated by organotins (TBT, TPT). The induction of imposex in marine snails is a classic example of the RXR-dependent disruption by organotins at nanomolar levels [24,26,60,62,63,64]. Furthermore, the evolutionary conservation of RXR-dependent modulation by organotins was suggested among lophotrochozoans, vertebrates, and crustaceans [27,33,65]. Recently, an RXR-dependent signaling pathway was suggested to promote the changes in the fatty acid profiles of the rotifer *B. koreanus* exposed to TBT [40]. The Organisation for Economic Co-operation and Development (OECD) suggests in vitro assays as a level 2 approach to test and assess endocrine disruption by environmental contaminants and denotes NRs as key players in this framework [17]. As follows, we addressed if TBT was able to activate the isolated RXRs in order to verify if RXR exploitation by TBT was also conserved during metazoan evolution. Our results demonstrated that the interaction of TBT with RXR is conserved among Metazoa, with the exception of Placozoa (*T. adhaerens*) (Figure 6). Moreover, we found that the condition described in rotifers [40] is not unique, since the cnidarian *A. aurita* RXR lacks the key cysteine and was nonetheless activated by TBT. The bryozoan *B. neritina* RXRs, on the other hand, yielded puzzling results, as similar responses were found independently of the conservation status of the cysteine residue. However, the substitution of the threonine residue by a cysteine led to a drastic increase of *B. neritina* RXR1 responsiveness. Regarding *B. neritina* RXR2, the deletion of the amino acid insertion found within the ligand-binding pocket also improved the receptor’s ability to bind TBT, albeit with lower potency than the RXR1 Thr388Cys mutant. These observations suggest that other ligand–pocket interactions could accommodate TBT binding, given that some pocket environments seem favorable to TBT binding even in the absence of the key cysteine residue. Yet, although not always strictly required, in more structurally conserved pockets, the substitution by a cysteine residue seems to enhance this interaction. Altogether, the analyses presented here indicate that the structural remodeling of RXR LBD during evolution favored the interaction of TBT with the cysteine residue, promoting a stronger sensitivity to this organotin.

## 5. Conclusions

In this study, we screened multiple metazoan genomes for the occurrence of RXR. This NR is present in all of the examined species, although with some important differences. We found that the ability of 9cisRA and TBT to transactivate RXR is conserved in more phyla than previously described. Moreover, we demonstrated that receptors with highly conserved sequences have different responses to ligand binding, highlighting the importance of studies with a broader species sampling. Yet, we were able to identify more cases of unconventional modulation of RXR by the organotin TBT, emphasizing the conservation of a common mechanism of endocrine disruption by TBT in Metazoa.

## Figures and Tables

**Table 1:** List of sequences used for the phylogenetic analysis of retinoid X receptor (RXR) in metazoan lineages and the corresponding accession numbers.

Phylum	Species	Nuclear Receptor		Accession Number
Chordata	*Homo sapiens*	NR1B1, 2, 3	RARα, β, γ	NP_000955.1, NP_000956.2, NP_000957.1
		NR2A1, 2	HNF4α, γ	NP_000448.3, NP_004124.4
		NR2F1, 2	COUP-TFI, TFII	NP_005645.1, NP_066285.1
		NR2B1, 2, 3	RXRα, β, γ	NP_002948.1, NP_068811.1, NP_008848.1
	*Branchiostoma floridae*	NR2B4	RXR	AAM46151.1
	*Branchiostoma lanceolatum*	NR2B4	RXR	ANP24206.1
Mollusca	*Lottia gigantea*	NR2B4	RXR	ESO92876.1
	*Patella vulgata*	NR2B4	RXR	ALQ43971.1
	*Nucella lapillus*	NR2B4	RXR	ABS70715.1
	*Reishia clavigera*	NR2B4	RXR	AAU12572.1
	*Acanthochitona crinita*	NR2B4	RXR	QAX24918.1
Annelida	*Bonellia viridis*	NR2B4	RXR	Bvi_RXR^1^
	*Platynereis dumerilii*	NR2B4	RXR	AVR59237.1
	*Capitella teleta*	NR2B4	RXR	ELT93409.1
Phoronida	*Phoronopsis californica*	NR2B4	RXR	Pca_RXR^1^
Brachiopoda	*Megathiris detruncata*	NR2B4	RXR	Mde_RXR^1^
	*Lingula anatina*	NR2B4	RXR	XP_013412668.1
Bryozoa	*Bugula neritina*	NR2B4-1,-2	RXR1, 2	Bne_RXR1^1^, Bne_RXR2^1^
	*Membranipora membranacea*	NR2B4-1,-2	RXR1, 2	SRX1121923
Rotifera	*Brachionus koreanus*	NR2B4	RXR	ASL70628.1
	*Brachionus plicatilis*	NR2B4	RXR	ASL70592.1
	*Brachionus rotundiformis*	NR2B4	RXR	ASL70517.1
	*Brachionus calyciflorus*	NR2B4	RXR	ASL70559.1
Platyhelminthes	*Schistosoma japonicum*	NR2B4	RXR	AFP95235.1
	*Schistosoma haematobium*	NR2B4	RXR	XP_012793373.1
	*Schistosoma mansoni*	NR2B4-1,-2	RXR1, 2	XP_018645908.1, AAD45325.1
Priapulida	*Priapulus caudatus*	NR2B4	RXR	QFQ33540.1
	*Halicryptus spinulosus*	NR2B4	RXR	SRX1343820
Arthropoda	*Uca pugilator*	NR2B4	RXR	AAC32789.3
	*Daphnia magna*	NR2B4	RXR	ABF74729.1
Xenoturbellida	*Xenoturbella bocki*	NR2B4	RXR	Xbo_RXR ^1^
Placozoa	*Trichoplax adhaerens*	NR2B4	RXR	ATD53319.1
Cnidaria	*Aurelia aurita*	NR2B4	RXR	AGT42223.1
	*Tripedalia cystophora*	NR2B4	RXR	AAC80008.1

^1^ These sequences were isolated in this study.

## Supplementary Materials

The following are available online at https://www.mdpi.com/2218-273X/10/4/594/s1, Table S1: List of primers used to isolate *RXR* genes, Table S2: List of primers used to amplify *RXR* hinge and LBD regions to be cloned into pBIND expression vector, Table S3: Ratio between firefly and *Renilla* luciferase luminescent values.
