# Supplementary material for: Of Retinoids and Organotins: The Evolution of the Retinoid X Receptor in Metazoa
Source: Biomolecules. 2020 Apr 11;10(4):594. doi: 10.3390/biom10040594 (PMC7225927; doi:10.3390/biom10040594)
Supplement: Supplementary file 1 [file biomolecules-10-00594-s001.pdf]

Supplementary Material

Of Retinoids and Organotins: the Evolution of the Retinoid X Receptor in Metazoa

Elza Fonseca [1,2], Raquel Ruivo [1], Débora Borges [1], João N. Franco [1], Miguel M. Santos [1,2,*] and L. Filipe C. Castro [1,2,*]

[1] Interdisciplinary Centre of Marine and Environmental Research, University of Porto, 4450-208 Matosinhos, Portugal; fonseca.ess@gmail.com (E.F.); ruivo.raquel@gmail.com (R.R.); debora.borges@ciimar.up.pt (D.B.); joaonunofranco@gmail.com (J.N.F.)

[2] Department of Biology, Faculty of Sciences, University of Porto, 4169-007 Porto, Portugal

* Correspondence: filipe.castro@ciimar.up.pt (L.F.C.C.), santos@ciimar.up.pt (M.M.S.); Tel.: +351-223-401-800

Table S1. List of primers used to isolate *RXR* genes. F, forward; R, reverse.

Species	Use	Primer sequences (5′→ 3′)	
	Degenerate	F	GCAGCTGGTCACCCTGGTNGARTGGGC
		R	GGCAGCCCAGCTCGGTYTTRTCCAT
Phoronopsis californica	5′ RACE	R1	TACCAACGCCTGCTTGATGAGCACT
		R2	TGAAGTGAGGCACCCTTTTAGCCCA
	3′ RACE	F1	TGGGCTAAAAGGGTGCCTCACTTCA
		F2	GCAGGCGTTGGTACCATATTTGACAG
	Full ORF	F	GTGAAATGAAAAATGGATAGATC
		R	CTAACTGACAGGACTCGG
Megathiris detruncata	5′ RACE	R1	GAACATGTAAACCTGTAGCAAGCAGG
		R2	GCAATTAGGAGTTCATTCCAGCCAGC
		R3	TTCTTTCATCTCGACAGGCGTAGG
	3′ RACE	F1	CCTGCTTGCTACAGGTTTACATGTTC
		F2	CTGAACTGGTAGCTAAGATGAGAGAA
	Full ORF	F	ATGGGTACTATAACATGGGTA
		R	TCATGTTGTTGGGCTGGGA
Bonellia viridis	5′ RACE	R1	CGATCAGCAACTCATTCCAACCTGC
		R2	CCAGGCACTTCATGTAGCGGCAGTA
		R3	TTGCACCCTTCGCAGCTGTAAACAC
	Full ORF	F	ATGCACGGTAACAGACAGC
		R	TCATGTGTTGGTCTGGAGAG
Bugula neritina 1	Partial ORF	F	CGTGTCGAGATGACAAATGC
		R1	GTCGAAGATTGTCCCTACGC
		R2	CTATACTGCGCAAGGCAGGT
	5′ RACE	R1	GTCGACGTACTGAGAGTTGCTTGGCTC
		R2	TCTGTCTGTGCTGCTCAGTACAGCGTC
		R3	CACGCAGCATTTGTCATCTCGACAC
		R4	TGAGGTGAACCGAGGCTTGAGTGAG
	3′ RACE	F1	GAGCCAAGCAACTCTCAGTACGTCGAC
		F2	CAGATCCATGGCTGTTCAAGATGGAATAC
	Full ORF	F	CTAACCGTGAAATGAATGGTC
		R	TTATTGCAGAGGGGCTTCTAA
Bugula neritina 2	5′ RACE	R1	TGTTGGTGAGAGGGTCGGAGGCTAT
		R2	CCGTCTCCGTGACTGTTGGACTTTG
	3′ RACE	F1	AGCTCACCTGTCTCAAGGCCATCGT
		F2	CCGGCAGGTTTGCTAAGCTCCTTCT
	Full ORF	F	ATGGGGATGAATGGAGCG
		R	CTAAGAGAGGTGTGTGTGG
Xenoturbella bocki	Partial ORF	F	CAATCTGTGCTATTTGTGGTG
		R	CATCTCCATCAAGAACGAATC
	5′ RACE	R1	TCCACTCCCAGCTCAGCCTCTAGGA
		R2	CAGTGCTGGCAGCGATTTCTCTGTT

		R3	CCGGAGATGTTGTGAGTAAGAGTGG
	3′ RACE	F1	GAGTCCTCACAGAGCTCGTCGCAAA
		F2	ACTAACGCAGACGACCCCAGCAGAT
	Full ORF	F	CAGTACAATGAATCCCATAGG
		R	GTTAAGCCATCGTTGTTGTTG

Table S2. List of primers used to amplify *RXR* hinge and LBD regions to be cloned into pBIND expression vector. F, Forward; R, Reverse.

Species		Primer sequences (5′→ 3′)	Restriction Enzymes
Homo sapiens α	F	aattTCTAGAGCCGTGCAGGAGGAGCGGCA	XbaI
	R	aattGGTACCAGTCATTTGGTGCGGCGCCT	KpnI
Bonellia viridis	F	aaaGGATCCGACAGCGAGTCAAGGAGAA	BamHI
	R	aaaGGTACCTCATGTGTTGGTCTGGAGAG	KpnI
Phoronopsis californica	F	aaaGGATCCTGAGAGCGAGGGATGGTC	BamHI
	R	tgtTCTAGACTAACTGACAGGACTCGG	XbaI
Megathiris detruncata	F	aattGGATCCAGAGAGGCAAAGATGGAAAGG	BamHI
	R	aattGGTACCTCATGTTGTTGGGCTGGGAG	KpnI
Bugula neritina 1	F	atcgGGATCCACAGACGAAACACAGCTGAC	BamHI
	R	aattGCGGCCGCTTATTGCAGAGGGGCTTCTAA	NotI
Bugula neritina 2	F	cccTCTAGAGATCCAAAGTCCAACAGTCA	XbaI
	R	aaaGGTACCCTAAGAGAGGTGTGTGTGG	KpnI
Priapulus caudatus	F	aaaTCTAGACAGCGCGTGAAGGAGAAG	XbaI
	R	aaaGGTACCCTACTCGCTGTTGCTCTC	KpnI
Xenoturbella bocki	F	gagTCTAGACAGAGGCCGAAAGATAACA	XbaI
	R	aaaGGTACCTTAAGCCATCGTTGTTGTTGA	KpnI
Trichoplax adhaerens	F	atcGGATCCGCGTAAAGAATAGCACACCAA	BamHI
	R	aaaGGTACCTTAAGAACTGCCTGTTTCCAG	KpnI
Aurelia aurita	F	atcgTCTAGAGAGTCAACGAAAAGAAAAGCAG	XbaI
	R	atatGGTACCTTAAGGCTGCGTGACCTCC	KpnI

Table S3. Ratio between firefly and *Renilla* luciferase luminescent values.

	Condition	Mean	SEM
Homo sapiens	Control	0.890	0.047
	9cisRA	24.085	1.282
Megathiris detruncata	Control	0.225	0.041
	9cisRA	13.752	3.155
Bonellia viridis	Control	0.647	0.053
	9cisRA	4.911	0.188
Phoronopsis californica	Control	0.334	0.021
	9cisRA	5.647	0.832
Bugula neritina 1	Control	0.240	0.033
	9cisRA	3.390	0.884
Bugula neritina 2	Control	0.430	0.196
	9cisRA	0.601	0.239
Priapulus caudatus	Control	0.392	0.096
	9cisRA	0.839	0.263
Xenoturbella bocki	Control	0.238	0.025
	9cisRA	0.431	0.042
Trichoplax adhaerens	Control	0.969	0.216
	9cisRA	21.092	8.742
Aurelia aurita	Control	0.369	0.065
	9cisRA	0.876	0.121
